# E-Gastryal^®^ + Magnesium Alginate Plus PPI vs. PPI Alone in GERD: Results from the GENYAL^®^ Randomized Controlled Trial

**DOI:** 10.3390/jcm14134794

**Published:** 2025-07-07

**Authors:** Cristiano Spada, Daniele Salvi, Silvia Pecere, Francesca Mangiola, Simone Varca, Serban Rosu, Vora Prateek, Petru Vasile Ciobanca, Adrian Goldis, Dionisio Franco Barattini, Guido Costamagna

**Affiliations:** 1 Digestive Endoscopy Unit, Fondazione Policlinico Universitario Agostino Gemelli IRCCS, 00168 Rome, Italy; cristiano.spada@policlinicogemelli.it (C.S.); silvia.pecere@policlinicogemelli.it (S.P.); francesca.mangiola@policlinicogemelli.it (F.M.); simone.varca01@icatt.it (S.V.); guido.costamagna@policlinicogemelli.it (G.C.); 2 Department of Translational Medicine and Surgery, Università Cattolica del Sacro Cuore, 00168 Rome, Italy; 3 Department of Gastroenterology and Endoscopy, Fondazione Poliambulanza Istituto Ospedaliero, 25124 Brescia, Italy; 4 University of Medicine and Pharmacy Victor Babes, Piața Eftimie Murgu 2, 300041 Timisoara, Romania; serbanrosu@gmail.com; 5 Medlife SA, Bld. Eroilor de la Tisa 28, 300551 Timisoara, Romania; dr.prateek@yahoo.com; 6 Centrul Medical Salvosan Ciobanca, Str. Dumbrava 48, Bloc TCI, 45051 Zalau, Romania; salvosan@salvosan.ro; 7 Cabinet Particular Policlinic Algomed, Str. Blaga Lucian 4, 300002 Timisoara, Romania; dr.adrian.goldis@algomed-timisoara.ro; 8 Opera CRO, a TIGERMED Group Company, Str. Cozia 10, 300209 Timisoara, Romania; barattini@operacro.com

**Keywords:** medical device, proton-pump inhibitor, gastroesophageal reflux disease, sodium alginate, mucosal protective agent

## Abstract

**Background**: Up to one-third of patients with gastroesophageal reflux disease (GERD) have persistent symptoms despite proton-pump inhibitor (PPI) therapy. E-Gastryal^®^ + MgAlg (Aurora Biofarma, Italy) is a mucosal protective agent that enhances barrier function against acid and non-acidic reflux. This study assessed its efficacy in combination with omeprazole versus omeprazole alone and as maintenance therapy. **Methods**: Patients with symptomatic GERD and Grade A reflux esophagitis confirmed by endoscopy were randomized to receive omeprazole 20 mg plus E-Gastryal^®^ + MgAlg or omeprazole 20 mg alone. The primary endpoint was the number of rescue medications used over 28 days. Secondary endpoints included symptom relief and quality-of-life assessments using the Reflux Symptom Index (RSI), Gastroesophageal Reflux Disease Impact Scale (GIS), GERD-Health-Related Quality of Life (GERD-HRQL), and Global Assessment of Performance (IGAP). **Results**: Ninety-six patients were included. The combination group used significantly fewer rescue medications (mean: 21 vs. 40.9 tablets; *p* = 0.002). At week 4, the combination group showed greater improvement in RSI, GIS, and GERD-HRQL scores (*p* < 0.001). Symptom relief was sustained during weeks 5–26 with E-Gastryal^®^ + MgAlg alone. **Conclusions**: E-Gastryal^®^ + MgAlg combined with omeprazole improves symptom control compared to PPI monotherapy. Continued use as maintenance therapy supports its role in long-term GERD management (NCT04130659).

## 1. Introduction

Gastroesophageal reflux disease (GERD) is a highly prevalent gastrointestinal disorder, affecting 4.16–22.4% of the global population [1]. It presents with typical symptoms, such as heartburn, chest pain, and regurgitation, and atypical manifestations, including chronic cough, hoarseness, and globus sensation [2]. GERD management includes lifestyle modifications, pharmacological interventions, and, in refractory cases, endoscopic or surgical treatment [3,4,5]. Proton-pump inhibitors (PPIs) are the first-line pharmacologic therapy, but approximately one-third of patients exhibit incomplete symptom resolution [6,7]. Moreover, prolonged PPI use is associated with potential adverse effects such as hypochlorhydria, hypergastrinemia, increased enteric infections, and suboptimal adherence may further reduce efficacy [8,9,10,11].

Up to 45% of GERD patients continue to experience symptoms despite PPI therapy, highlighting the need for adjunctive treatments [12,13,14,15]. This challenge has driven growing interest in complementary therapies, which have traditionally included alginates, antacids, or non-specific gastric mucosal protective agents, such as acid–haluronic and sulphate-based bioadhesive formulations [16,17,18].

In recent years, multiple medical devices and pharmacological formulations have been developed to optimize GERD management, potentially representing a significant advancement in the treatment of both GERD and laryngopharyngeal reflux (LPR) symptoms [19]. Among these, E-Gastryal^®^ + MgAlg, a combination of hyaluronic acid, hydrolyzed keratin, tara gum, xanthan gum, and magnesium alginate, has been introduced as a novel mucosal protective agent. An observational study in Italy involving over 1800 GERD patients treated with PPIs demonstrated significant symptom reduction in those receiving E-Gastryal^®^ + MgAlg as an adjunct therapy [20,21].

The primary aim of this study (GENYAL^®^) was to assess whether a one-month treatment with E-Gastryal^®^ + MgAlg, in combination with omeprazole, reduces the total consumption of alginate-based rescue therapy. Additionally, this study evaluated the efficacy of E-Gastryal^®^ + MgAlg as a maintenance therapy by analyzing its impact on erosive GERD symptoms over a subsequent five-month period.

## 2. Materials and Methods

### 2.1. Study Design

GENYAL is an open-label, parallel-group, controlled, multi-center, randomized study consisting of a 28-day comparative treatment phase followed by a five-month non-comparative follow-up period.

This study was conducted in accordance with the Declaration of Helsinki (7th revision, Brazil, October 2013) and adhered to the International Conference on Harmonization (ICH) Good Clinical Practice (GCP) guidelines, as well as the Medical Devices guidelines (MEDDEV). The trial was registered in the EudraCT database (EudraCT No. 2019-004062-17) and listed on ClinicalTrials.gov (NCT04130659). Written informed consent and a signed General Data Protection Regulation (GDPR) statement were obtained from all enrolled participants. The reporting of this study complies with the CONSORT guidelines for clinical trials [22].

### 2.2. Study Participants

The five centers participating in this study were selected based on their expertise in managing gastroesophageal reflux disease (GERD). Participants were evaluated for eligibility at a screening visit conducted one week before baseline. Eligible patients were adults (aged 18–65 years) of both sexes, diagnosed with GERD (Grade A reflux esophagitis on Los Angeles Classification System [23]) confirmed by a gastroscopy performed within one month before baseline, and reporting episodic heartburn and/or acid regurgitation at least three times per week in the two weeks before baseline. Patients were not supposed to have taken any specific therapy for GERD in the two weeks before baseline. A body mass index (BMI) of 18.5–36 kg/m^2^ and the ability to provide written informed consent were also required.

Patients were excluded if they met any of the following criteria: (i) history of esophageal disease, active gastric/duodenal ulcers within the past three months, or acute upper gastrointestinal bleeding within six months prior to baseline; (ii) prior surgery involving the esophagus or upper gastrointestinal tract; (iii) severe renal or hepatic insufficiency; (iv) use of PPIs, E-Gastryal^®^ + MgAlg, systemic glucocorticoids, or medications that could interfere with study drugs (e.g., theophylline, warfarin, and/or phenytoin), or NSAIDs (except enteric-coated aspirin ≤150 mg/day) within 28 days prior to baseline; (v) pregnancy, lactation, or lack of reliable contraception in females of childbearing potential; (vi) history of drug or alcohol abuse within 12 months prior to baseline; (vii) clinically significant laboratory abnormalities or comorbidities that, in the investigator’s opinion, could pose a risk to the patient or interfere with study results; (viii) known hypersensitivity to omeprazole, E-Gastryal^®^ + MgAlg, or sodium alginate or sodium bicarbonate; and (ix) participation in another trial within 30 days before baseline.

Between 8 August 2019 (first patient, first visit) and 22 March 2023 (last patient, last visit), 100 patients were enrolled, and 96 were included in the analysis.

### 2.3. Interventions and Allocation

Randomization was performed centrally by the Contract Research Organization managing this study (Opera CRO—Timisoara, Romania) using a permuted block of random sizes. The package was GNU Library General Public License, v 2.1, from R statistical software v 3.5. [24,25]. Patients were allocated a unique 5-digit patient randomization number using an online web service. Patients were randomized (1:1) into two treatment groups: Group A E-Gastryal^®^ + MgAlg (Marial^®^, Nekkar Lab, Milan, Italy), administered as one stick twice daily after meals from day 1 to day 28, plus omeprazole (20 mg, Aurobindo Pharma, Saronno, Italy) once daily; and Group B Omeprazole (20 mg, Aurobindo Pharma, Saronno, Italy) once daily from day 1 to day 28. Patients and investigators were not blinded to the allocated treatment.

During the initial 28-day study period, patients in both groups were permitted to self-administer a rescue medication containing sodium alginate (250 mg), sodium hydrogen carbonate (133.5 mg), and calcium carbonate (80 mg) (Reckitt Benckiser Healthcare, Milan, Italy) for symptom relief when necessary. Rescue medication use was recorded and included in the analysis.

At the end of the 28-day treatment phase, eligible patients entered the five-month follow-up period (days 29–180). During this phase, all participants received E-Gastryal^®^ + MgAlg alone (one stick twice daily after meals) as maintenance therapy. Study medications were provided by the Sponsor.

### 2.4. Assessments and Outcomes

The primary outcome was the total consumption of alginate-based rescue therapy during the 28-day treatment phase in both groups. The choice of this parameter as primary outcome was approved by the Ethics Committee in Romania (2 August 2019) and in Italy 20 February 2020). It was assessed by reviewing patient diaries and calculating the number of unused blister packs initially provided at baseline.

Secondary outcomes were changes in Reflux Symptoms Index (RSI), GERD Impact Scale (GIS), GERD Health-Related Quality of Life (GERD-HRQL), and Global Assessment of the Performance (IGAP) scores during the first and follow-up periods of this study. Quality of Life and Symptoms scale tests (RSI, GIS, and GERD-HRQL-PGAS) were performed at visit 1 (baseline), 2, 3, and 4 (follow-up visit at 180 days). IGAP was evaluated at visits 3 and 4.

RSI is a nine-item self-administered questionnaire assessing LPR symptoms. Each item is scored from 0 (no problem) to 5 (severe problem), with a total score > 13 indicating LPR [24]. GIS is a nine-question patient-reported instrument assessing symptom frequency over the past weeks, using a four-point scale (1 = none of the time to 4 = all of the time) [26]. GERD-HRQL is a 10-question instrument measuring symptom severity and impact on daily life, with scores ranging from 1 to 5 (maximum total: 50). Higher scores indicate greater symptom burden [27]. IGAP scale is a four-point scale (1 = excellent, 2 = good, 3 = moderate, and 4 = poor) assessed at days 28 and 180.

Safety was evaluated throughout this study by monitoring adverse events (AEs), serious adverse events (SAEs), adverse device effects (ADEs), and serious adverse device effects (SADEs). Investigators also used the four-point Investigator Global Assessment of Safety (IGAS) scale (1 = excellent, 2 = good, 3 = fair, and 4 = poor) to assess overall treatment safety at days 28 and 180.

### 2.5. Monitoring and Data Management

All study data were recorded in an electronic case report form (eCRF), which was securely accessible only to authorized personnel. Investigators were responsible for data entry and approval. Monitoring activities were conducted by the CRO according to its Standard Operating Procedures (SOPs). Remote monitoring and real-time data review were performed via the eCRF, while on-site monitoring visits were conducted to verify source documents (e.g., medical charts and informed consent forms). The electronic data capture system complied HIPAA, BAA, and GDPR. Data storage services were verified for SSAE 18 SOC 1 and 2 compliance and met EU data residency requirements.

### 2.6. Sample Size

The success rate in reducing the self-administration of a typical symptomatic OTC (Gaviscon^®^) used as rescue medication in a population affected by GERD was indicated as the primary outcome for sample size calculation. The consumption of the rescue medication should be compared between the two arms in the comparative part of this study (from baseline to week 4). In the original study protocol, the investigators specified that the sample size calculation would employ a strong conservative approach, thereby exceeding the recently published positive results of the tested medical device [28]. In addition, this article did not report the confidence interval, precluding the estimation of precision for the effect. Using the raw success rates, we calculated the effect size as Cohen’s h = 0.60, which indicates a medium-to-large effect [29]. The calculation of the sample size was conducted under the assumption of an α = 0.05 (two-sided), 80 % power, and an expected absolute difference of 26% (0.60 to 0.86). The two-proportion test (Fisher’s exact in analysis, normal approximation for power) was utilized, resulting in 45 patients per group. Adopting a conservative approach, the total number of participants was rounded to 100, with 50 subjects per arm. An amendment was presented by the Sponsor and approved by the EC for completing this study on June 2024, with a total of 100 patients enrolled. This was motivated by the recent interim analysis of the study population enrolled in Romania, which evidenced a statistical difference between the two groups in the primary outcome, confirming the overestimation of the initial sample size. The Italian Ethics Committee authorized the amendment and premature termination of this study after the enrollment of 100 patients on 25 July 2024.

### 2.7. Statistical Analyses

All statistical analyses were performed using the R statistical software v 3.5. The overall type I error rate was preserved at 5%. All *p*-values are two-sided. Continuous variables with a normal distribution (assessed via Shapiro–Wilk test and Q–Q plots) are reported as mean ± SD; non-normal variables are presented as median (IQR). Categorical data are summarized as counts and percentages. The Student’s *t*-test and the Mann–Whitney test were employed to perform comparative analysis in accordance with the distribution of these variables. Categorical variables were compared between groups using either the Chi-square test or Fisher’s Exact Test, depending on the distribution of the data. Specifically, the Chi-square test was applied when all expected cell counts were ≥5, while Fisher’s Exact Test was used when this assumption was violated, to ensure accurate *p*-values in cases of small sample sizes or sparse data. To assess the effects of group (between-subjects factor), time (within-subjects factor), and their interaction on continuous outcomes, a repeated-measures General Linear Model (GLM) was applied. This model accommodates both between- and within-subjects factors and allows for testing main and interaction effects. Assumptions of normality, homogeneity of variance, and sphericity were evaluated, with Greenhouse–Geisser corrections applied where necessary.

## 3. Results

### 3.1. Patient Disposition and Baseline Characteristics

**Figure 1:**
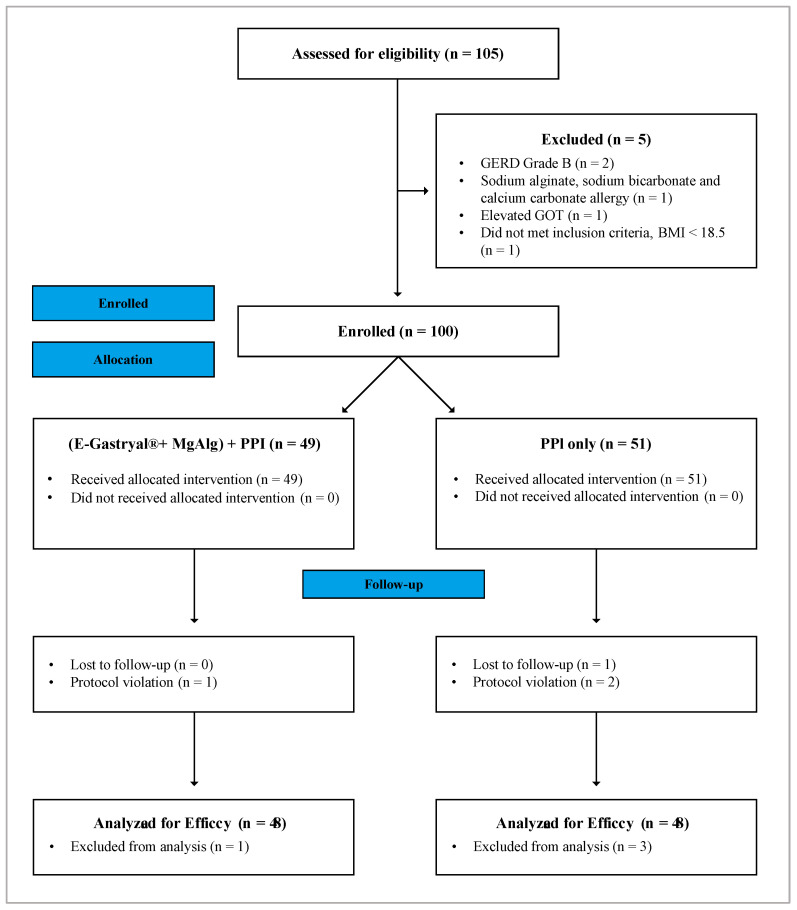
CONSORT flow diagram.

A total of 105 patients were screened, with 100 meeting eligibility criteria and undergoing randomization. Four patients were excluded due to protocol violations (N = 3) or loss of follow-up (N = 1), while one patient withdrew due to adverse events. Consequently, 96 patients (45 males and 51 females) completed this study per protocol (Figure 1). The mean age at enrollment was 43.1 years (SD: 11.3, range: 18–65). The Intent-To-Treat (ITT) and safety populations were identical. Demographic and baseline characteristics were comparable between treatment groups (Table 1).

### 3.2. Primary Outcome: Rescue Medication Use

Patients in the E-Gastryal^®^ + MgAlg + omeprazole group required significantly fewer rescue medication tablets over the 28-day study period compared to the omeprazole-alone group (*p* = 0.002, Mann–Whitney test) (Table 2).

**Figure 2:**
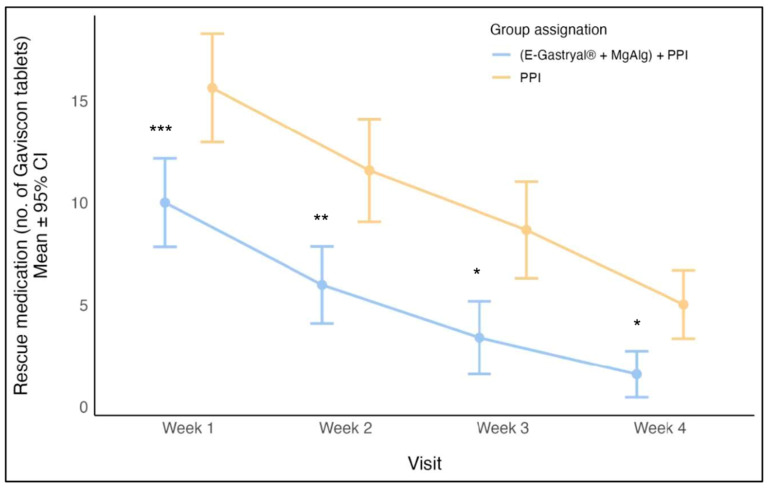
Rescue medicine (mean ± 95% CI) usage by study visits and group assignment in the first 28 days of study. *: *p* = 0.001; **: *p* = 0.002; ***: *p* = 0.003, Mann–Whitney test with Bonferroni multiplicity correction.

The analyses of weekly rescue medication consumption confirmed this trend, with statistically significant differences observed between the two treatment groups when performing a Mann–Whitney test with Bonferroni multiplicity correction each week (week 1, *p* = 0.003, 95% CI: 2.0–9.0; week 2, *p* = 0.002, *p* = 0.003, 95% CI: 2.0–9.0; weeks 3, *p* = 0.001, 95% CI: 6.6–9.0; week 4, *p* = 0.001, 95% CI: 4.0–8.3) (Figure 2). A GLM statistical test was used to identify the predictors (independent variables) that influenced the total amount of rescue medication (the dependent variable) taken during the four-week period. The analysis identified baseline BMI as a significant predictor of rescue medication use. Specifically, a one-unit increase in BMI correlated with an average increase of 1.74 rescue tablets by week four. The GERD-HRQL score at baseline also showed a positive association with increased use of rescue medications (mean increase: 0.94 tablets). Assignment to the E-Gastryal^®^ + MgAlg + omeprazole group, meanwhile, was associated with a mean reduction of 20.5 tablets. Other factors, including GIS, RSI, and gender, were not correlated with the number of rescue medications used during the four-week study period.

### 3.3. Secondary Outcomes

**Figure 3:**
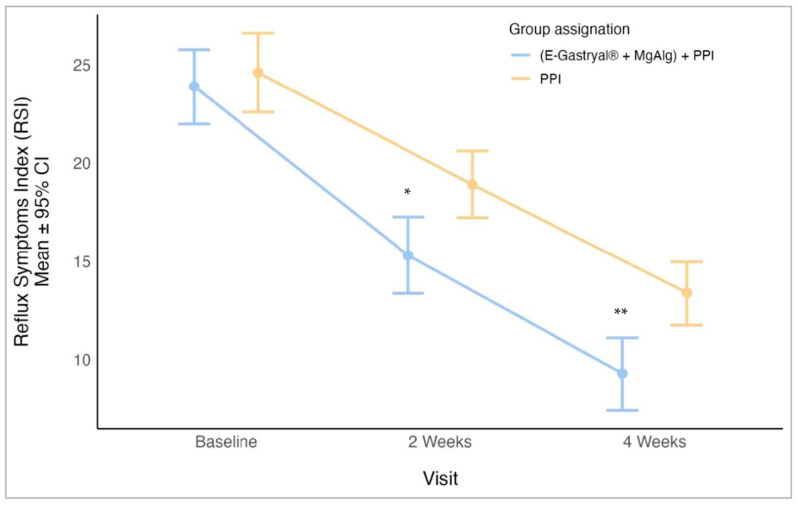
Reflux Symptoms Index (RSI) (mean ± 95% CI) by study visits and group assignment in the first 28 days of study. *: *p* = 0.011; **: *p* = 0.001, Mann–Whitney test with Bonferroni multiplicity correction.

RSI scores demonstrated a statistically significant improvement in the E-Gastryal^®^ + MgAlg + omeprazole group compared to the omeprazole-alone group, as evidenced by a Mann–Whitney test with Bonferroni multiplicity correction at weeks 2 (*p* = 0.011, 95% CI: 1.0–7.0) and 4 (*p* < 0.001, 95% CI: 1.0–7.0) (Figure 3). The improvement persisted during the follow-up period, with mean RSI scores decreasing from 11.3 ± 6.40 at day 28 to 2.8 ± 4.67 at day 180 (*p* < 0.001) (Figure S1).

**Figure 4:**
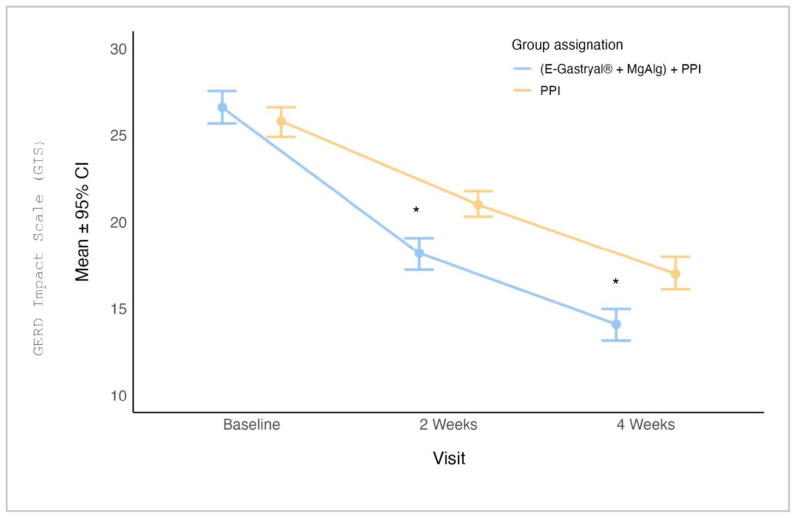
GERD Impact Scale (GIS) Patient’s Questionnaire (mean ± 95% CI) by study visits and group assignment in the first 28 days of study. *: *p* = 0.001, Mann–Whitney test with Bonferroni multiplicity correction.

The results of the GIS revealed that the E-Gastryal^®^ + MgAlg + omeprazole group exhibited significantly better scores at weeks 2 (*p* < 0.001, 95% CI: 1.0–4.0) and 4 (*p* < 0.001, 95% CI: 2.0–5.0). (Figure 4). The benefit persisted through day 180, with mean GIS scores declining from 15.6 ± 3.42 (day 28) to 10.6 ± 3.06 (day 180) (*p* < 0.001) (Figure S2).

**Figure 5:**
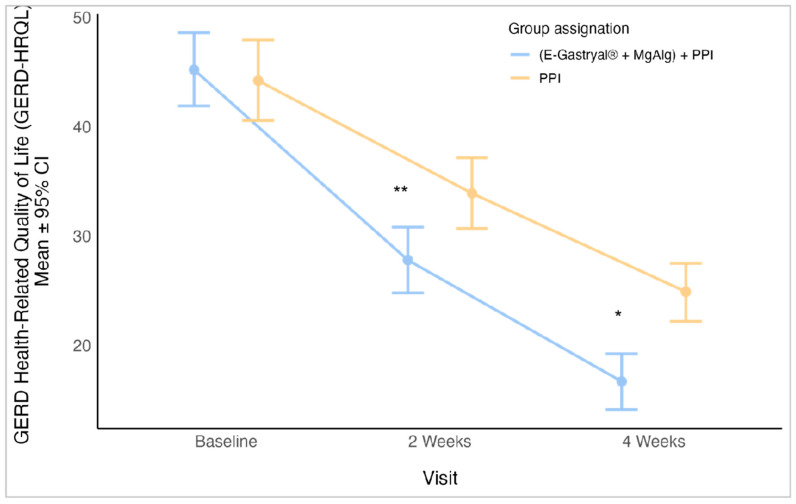
GERD Health-Related Quality of Life (GERD-HRQL) Patient’s Questionnaire (mean ± 95% CI) by study visits and group assignment in the first 28 days of study. *: *p* = 0.001, **: *p* = 0.003, Mann-Whitney test with Bonferroni multiplicity correction.

A statistically significant decrease in GERD-HRQL scores was observed in the E-Gastryal^®^ + MgAlg + omeprazole group (*p* = 0.003, 95% CI: −2.0–11.0) at week 2 and (*p* < 0.001, 95% CI: −4.0–13.0) at week 4 (Figure 5). The trend continued into follow-up, with scores decreasing from 20.8 ± 9.9 (day 28) to 4.1 ± 6.4 (day 180) (*p* < 0.001) (Figure S3).

At day 28, investigators rated the treatment as “excellent” in 56.3% of E-Gastryal^®^ + MgAlg + omeprazole patients versus 16.6% in the omeprazole-alone group (*p* < 0.001, Fisher’s test). No significant difference in GERD recurrence was observed at day 180 between groups (*p* = 0.653, Chi-square test).

### 3.4. Safety

Six patients reported adverse events (four in the E-Gastryal^®^ + MgAlg + omeprazole group; two in the omeprazole-alone group). One patient belonging to the omeprazole group withdrew due to moderate abdominal cramps and dermatitis, possibly treatment-related. Four patients reported gastric burning, deemed moderate and unlikely related to treatment. The event was, in any case, considered as moderate and unlikely related to the study treatment; no actions were taken, and the patient continued the study treatment. One patient in the omeprazole-alone group experienced a transient sweet taste sensation. The event was considered as possibly related to concomitant food consumption and judged as mild; no actions were taken, and the patient continued the study treatment. No serious adverse events occurred (Table 3).

## 4. Discussion

A 4-week treatment with E-Gastryal^®^ + MgAlg in association with omeprazole provides better control of GERD-related symptoms compared to omeprazole monotherapy. This conclusion is supported by the significantly lower consumption of rescue medication tablets in the combination therapy group during the initial 28-day period, suggesting that E-Gastryal^®^ + MgAlg enhances the efficacy of acid suppression therapy. These findings reinforce the relevance of adjunctive therapies in GERD management, particularly for patients experiencing incomplete symptom relief with PPIs alone [30].

The observed difference in rescue medication use underscores a well-documented limitation of PPI therapy: its inability to fully control symptoms in a subset of GERD patients. The reasons behind this therapeutic gap are multifaceted and may include patient-related factors such as inconsistent adherence, physician-related elements like misdiagnosis, and drug-related considerations, including inadequate duration of action [13]. Effective GERD management requires a multifactorial approach that not only suppresses acid production but also minimizes the duration and intensity of acid exposure in the esophageal mucosa. Mucosal protective agents play a pivotal role in this context by creating a protective layer that mitigates irritation, accelerates mucosal repair, and reduces symptom severity. Although symptom control, assessed through rescue medication use, represents a subjective endpoint, it was selected for its clinical relevance and its capacity to objectively reflect patient-perceived treatment efficacy.

Alginates contribute to GERD therapy by forming a physical barrier, a raft, that isolates the acid pocket, a region of unneutralized gastric acid that accumulates postprandially in the proximal stomach [7]. This mechanism prevents acid migration into the esophagus, a key factor in symptom persistence and disease progression. Alginate-based therapies increased the odds of resolution of GERD symptoms when compared to placebo or antacids [31]. The results of the GENYAL^®^ study align with this concept, demonstrating that E-Gastryal^®^ + MgAlg, when combined with omeprazole, significantly alleviates GERD symptoms by limiting acid exposure in the distal esophagus and promoting mucosal protection. The benefits in the present study were evaluated in patients with erosive disease, where enhanced mucosal defense mechanisms likely contributed to improved healing outcomes.

Notably, the symptomatic improvements observed in the combination therapy group were sustained beyond the initial treatment phase. During the follow-up period (weeks 5–26), patients who continued E-Gastryal^®^ + MgAlg as monotherapy maintained symptom relief, suggesting its efficacy in reducing acid, bile, and other luminal irritants even in the absence of PPI therapy. The sustained reductions in RSI and GIS scores (*p* < 0.001) across the study period support current GERD treatment strategies that advocate for initial acid suppression followed by maintenance therapy with mucosal protectants. The long-term control of GERD-related symptoms of E-Gastryal^®^ + MgAlg when used as monotherapy following an initial treatment with PPI may suggest a potential role of E-Gastryal^®^ + MgAlg as maintenance treatment, potentially reducing PPI prescription. This hypothesis needs to be confirmed in large prospective randomized trials specifically designed to evaluate the performance of E-Gastryal^®^ + MgAlg when used as maintenance treatment as compared to PPI. The long-term efficacy of the maintenance follow-up needs to be underlined. Several factors may be responsible for the variation in the response of GERD patients, including individual variability, diet, and type of reflux (acid vs. non-acid). Notably, during the follow-up period, no additional pharmacological interventions (e.g., prokinetics or PPIs) were permitted, thereby allowing the evaluation of E-Gastryal^®^ + MgAlg as a standalone therapy. The observed efficacy across different GERD phenotypes suggests that this formulation may offer symptom relief independent of reflux type.

Regarding safety, six patients reported mild-to-moderate adverse events during the study period: four in the E-Gastryal^®^ + MgAlg plus omeprazole group and two in the omeprazole-only group. The adverse events primarily consisted of gastrointestinal discomfort. One patient in the omeprazole group withdrew due to adverse effects, including mild-to-moderate abdominal cramps and moderate dermatitis. In all remaining cases, adverse events were mild, required no medical intervention, and did not necessitate treatment discontinuation, further supporting the favorable safety profile of E-Gastryal^®^ + MgAlg.

This study presents major strengths. A homogeneous sample of GERD patients with typical symptoms and Grade A esophagitis (according to the Los Angeles classification at endoscopy) was included. Although according to the recently updated version of the Lyon Consensus, Grade A esophagitis is not conclusive for a diagnosis of GERD [2], the combination of endoscopic features and GERD-related symptoms was used to ensure the inclusion of a real GERD patient population. Symptom variations were systematically assessed using validated quality-of-life scoring systems, including the RSI, GERD-HRQL questionnaire, GIS, and IGAP, administered before and after treatment. The results reinforce previous observational data from a multi-center study across 56 Italian gastroenterology centers, confirming the efficacy and safety of this medical device [20,21].

The present study has intrinsic limitations. A functional test (i.e., pH-impedance monitoring) was not performed. Although the pH-impedance would have provided information regarding the underlying pathophysiology, this invasive diagnostic procedure is rarely performed in general practice nor is it recommended in the initial evaluation of patients suffering from GERD. This study does not provide any information regarding mucosal healing since an endoscopic follow-up was not performed; therefore, differences between the two groups in terms of the reduction or disappearance of epithelial lesions were not evaluated. Future research should explore the long-term clinical significance of E-Gastryal^®^ + MgAlg, particularly in terms of esophageal tissue repair and regeneration. Moreover, this study does not provide any information regarding conclusive GERD-related erosive esophagitis (Grade B, C, and D according to Los Angeles classification), highlighting the need for further investigations in this subset of patients. Lastly, the absence of blinding represents a limitation of this RCT; however, potential bias was mitigated through the use of objective outcome measures and standardized procedures.

In conclusion, the findings from this study confirm the therapeutic potential of E-Gastryal^®^ + MgAlg in GERD management, providing an improved control of GERD-related symptoms, particularly as an adjunct to PPI therapy. Its ability to provide both acute and sustained symptom relief suggests that it may serve as an effective maintenance therapy following initial acid suppression. Future research should explore its long-term impact on mucosal integrity and its potential to reduce the use of PPIs in chronic GERD symptoms management. 

## Figures and Tables

**Table 1:** Demographic characteristics of all subjects included in the PP population at baseline.

Characteristic	Group	Category	N	Mean (SD)	Median	Min	Max
Age (years)	E-Gastryal^®^ + MgAlg + omeprazole	Female	23	45.5 (11.91)	48	23	62
		Male	25	42.3 (10.33)	42	18	64
		All	48	43.8 (11.12)	44.5	18	64
	omeprazole alone	Female	28	42.0 (11.65)	42	20	64
		Male	20	42.9 (11.48)	42	24	65
		All	48	42.4 (11.47)	42	20	65
Weight (kg)	E-Gastryal^®^ + MgAlg + omeprazole	Female	23	67.1 (16.14)	64	48	101
		Male	25	83.3 (13.85)	87	60	110
		All	48	75.5 (16.94)	76	48	110
	omeprazole alone	Female	28	64.4 (11.46)	62	46	95
		Male	20	83.2 (10.90)	83	60	103
		All	48	72.3 (14.53)	70.5	46	103
Height (cm)	E-Gastryal^®^ + MgAlg + omeprazole	Female	23	164.3 (7.02)	165	148	178
		Male	25	175.2 (5.77)	175	164	186
		All	48	170.0 (8.38)	170	148	186
	omeprazole alone	Female	28	166.4 (6.15)	166.5	154	178
		Male	20	176.0 (7.93)	173.5	165	198
		All	48	170.4 (8.38)	170	154	198
Body Mass Index (kg/m^2^)	E-Gastryal^®^ + MgAlg + omeprazole	Female	23	24.6 (4.65)	24.2	18.7	35.8
		Male	25	27.1 (4.24)	27.5	21.3	35.9
		All	48	25.9 (4.56)	25.5	18.7	35.9
	omeprazole alone	Female	28	23.3 (4.09)	22.1	18.8	34.9
		Male	20	26.9 (3.35)	26.9	21.9	34.8
		All	48	24.8 (4.17)	23.9	18.8	34.9

**Table 2:** Number of rescue (alginate) tablets used by patients during the first 28 days of this study. A statistically significant difference between the two groups was found using the Mann–Whitney U test (*p* = 0.002).

Group	Gender	Patients	Rescue Medication (Tablets)	Mean (SD)	Median	Minimum	Maximum
E-Gastryal^®^ + MgAlg + omeprazole	Female	23	356	15.5 (15.1)	14	0	56
	Male	25	651	26.0 (26.1)	20	0	86
	All	48	1007	21.0 (22.0)	16.5	0	86
omeprazole alone	Female	28	1088	38.9 (29.3)	45	0	89
	Male	19	833	43.8 (32.2)	41	0	96
	All	47	1921	40.9 (30.3)	42	0	96

**Table 3:** Comparison between the two treatment groups by Investigator Global Assessment of Safety (IGAS) during the first 28 days. Changes in IGAS scores during the second part of this study (from day 28 to day 180) when all patients were treated with E-Gastryal^®^ + MgAlg. IGAS was reported on a 4-point scale: 1 = excellent safety, 2 = good safety, 3 = moderate safety, and 4 = poor safety.

Group		N	Excellent	Good	Moderate	Poor
Day 1–28	E-Gastryal^®^ + MgAlg + PPI	48 (100%)	31 (66.3%)	17 (36.2%)	0 (0.0%)	0 (0.0%)
	PPI	49 (100%)	33 (67.3%)	16 (32.7%)	0 (0.0%)	0 (0.0%)
Day 28–180	E-Gastryal^®^ + MgAlg	95 (100.0%)	80 (84.2%)	15 (15.8%)	0 (0.0%)	0 (0.0%)

## Data Availability

The raw data supporting the conclusions of this article will be made available by the authors upon request.

## Supplementary Materials

The following supporting information can be downloaded at: https://www.mdpi.com/article/10.3390/jcm14134794/s1, Figure S1: Mean ±95% CI Reflux Symptoms Index (RSI) Patient’s Questionnaire by study visits and group assignation at week 4 and week 25. Figure S2: Mean ±95% CI GERD Impact Scale (GIS) Patient’s Questionnaire by study visits and group assignation at week 4 and week 25. Figure S3. Mean ±95% CI GERD Health-Related Quality of Life (GERD-HRQL) Patient’s Questionnaire by study visits and group assignation at week 4 and week 25.
