# Supplementary material for: E-Gastryal® + Magnesium Alginate Plus PPI vs. PPI Alone in GERD: Results from the GENYAL® Randomized Controlled Trial
Source: J Clin Med. 2025 Jul 7;14(13):4794. doi: 10.3390/jcm14134794 (PMC12251391; doi:10.3390/jcm14134794)
Supplement: Supplementary file 1 [file jcm-14-04794-s001.zip › jcm-3664470-supplementary.docx]

**TITLES OF FIGURES**


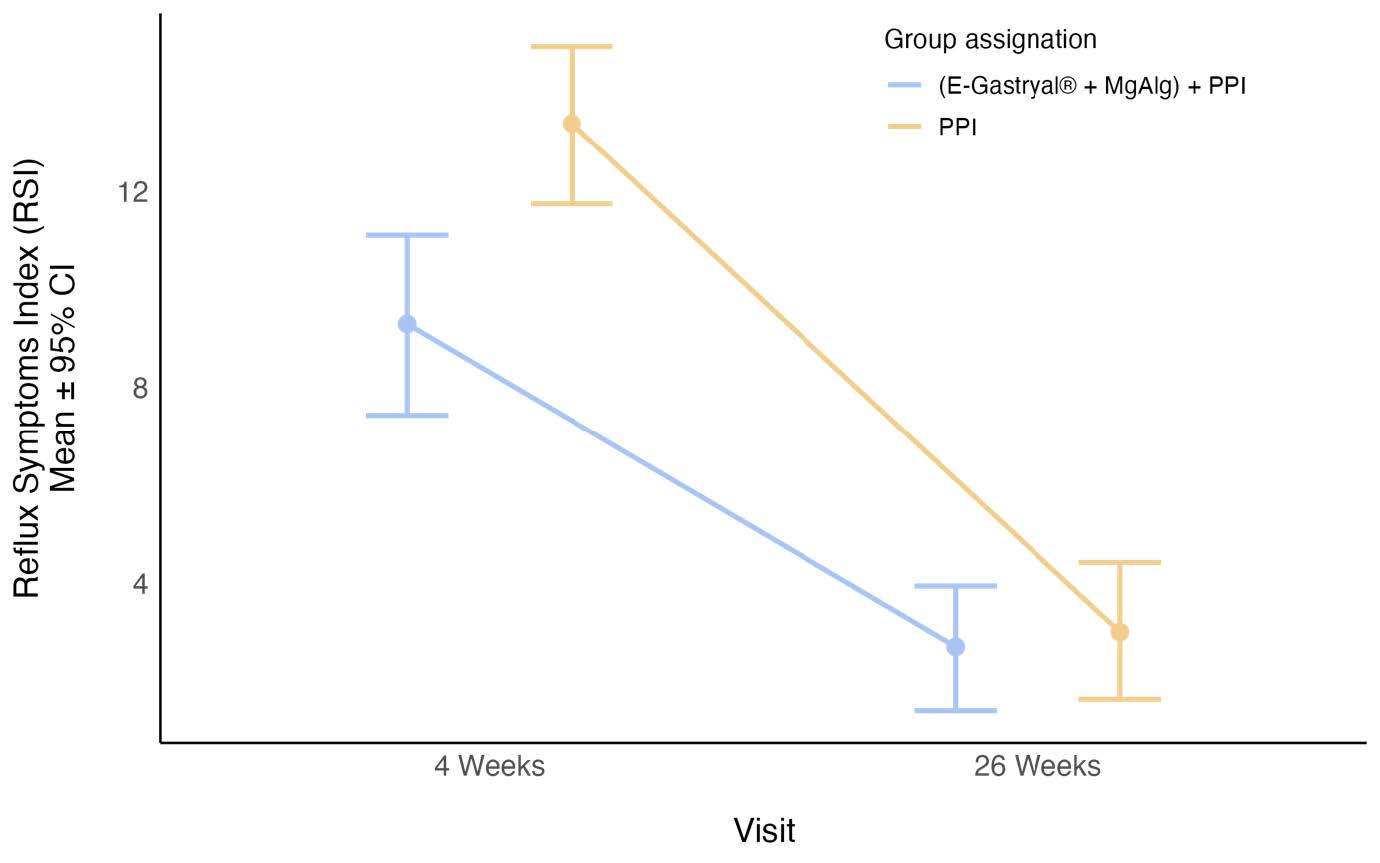
Figure 1S Mean ±95% CI Reflux Symptoms Index (RSI) Patient’s Questionnaire by study visits and group assignation at week 4 and week 25.


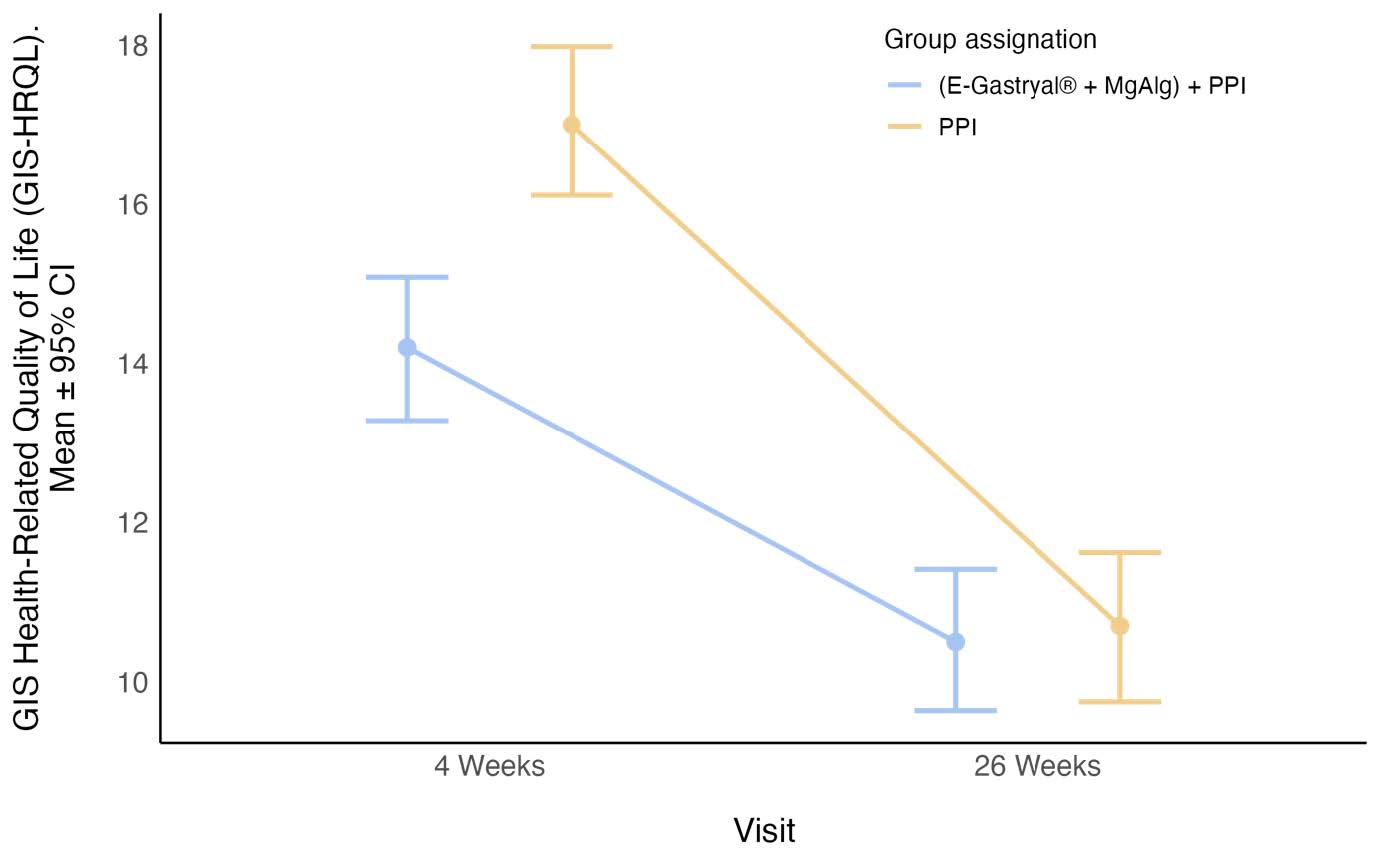
Figure 2S Mean ±95% CI GERD Impact Scale (GIS) Patient’s Questionnaire by study visits and group assignation at week 4 and week 25.


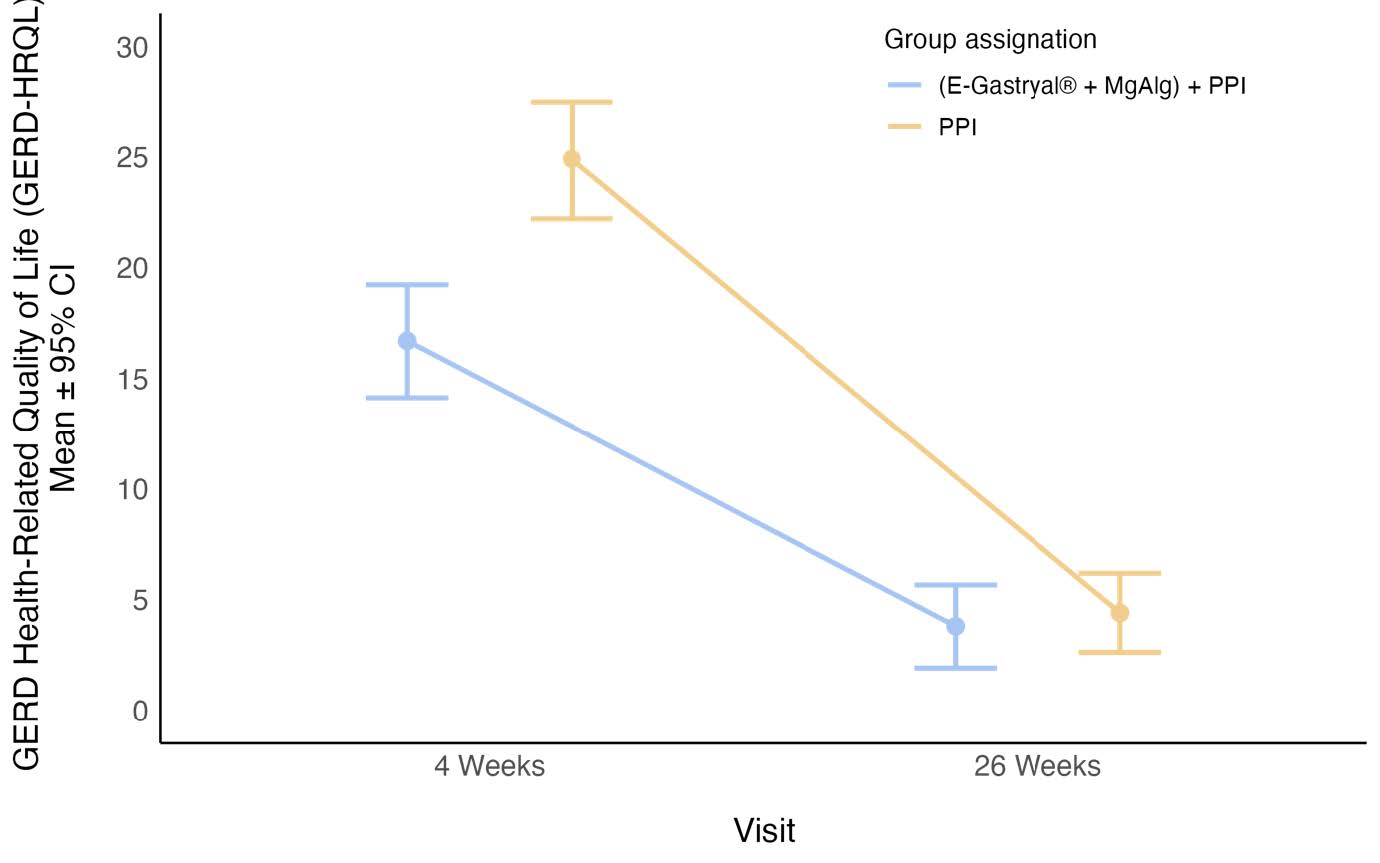
Figure 3S Mean ±95% CI GERD Health-Related Quality of Life (GERD-HRQL) Patient’s Questionnaire by study visits and group assignation at week 4 and week 25.
